# FAP-Directed Imaging and Therapy in Head and Neck Cancer of Unknown Primary

**DOI:** 10.3390/cancers17132205

**Published:** 2025-06-30

**Authors:** Sophie C. Kunte, Gabriel T. Sheikh, Frederik L. Giesel, Martin Canis, Rudolf A. Werner

**Affiliations:** 1 Department of Nuclear Medicine, LMU University Hospital, LMU Munich, 81377 Munich, Germany; 2 Bavarian Cancer Research Center (BZKF), Partner Site Munich, 81377 Munich, Germany; 3 Department of Nuclear Medicine, Medical Faculty of Heinrich Heine University, University Hospital Düsseldorf, 40225 Düsseldorf, Germany; 4 Department of Otorhinolaryngology, LMU University Hospital, LMU Munich, 81377 Munich, Germany; 5 The Russell H Morgan Department of Radiology and Radiological Sciences, Division of Nuclear Medicine, Johns Hopkins School of Medicine, 81377 Munich, Germany

**Keywords:** HNCUP, FAPI, PET/CT, diagnostic, theranostics

## Abstract

Cancer of unknown primary in the head and neck (HNCUP) region poses a major diagnostic and therapeutic challenge. While [^18^F]FDG positron emission tomography/computed tomography (PET/CT) is widely used for oncologic staging, it frequently fails to accurately identify the primary tumor in HNCUP cases, limiting its diagnostic accuracy and clinical utility. In contrast, Gallium-68 or F-18-labelled fibroblast activation protein inhibitor (FAPI) PET/CT has emerged as a promising alternative by selectively targeting cancer-associated fibroblasts, which are abundant in the tumor microenvironment. In recent years, FAPI-PET/CT has been increasingly investigated as a complementary or superior imaging modality to FDG-PET/CT for tumor detection in HNCUP, with potential implications for patient management. In a theranostic setting, patients with widespread disease demonstrating increased target expression on FAPI-PET have also been treated using Lutetium-177-labeled FAP-directed therapeutic equivalents. This review provides a comprehensive overview of the current evidence on FAPI-PET/CT in HNCUP, emphasizing its diagnostic advantages, potential impact on therapeutic decision making, and future directions for research in this evolving field, including FAP-directed radioligand therapies.

## Simple Summary

Head and neck cancer of unknown primary (HNCUP) poses significant diagnostic challenges, as [^18^F]FDG positron emission tomography/computed tomography (PET/CT) often fails to detect tumor. Fibroblast activation protein inhibitor (FAPI) PET/CT has emerged as a promising alternative by targeting the tumor microenvironment and has shown improved diagnostic potential. This review summarizes the current evidence on FAPI-PET in HNCUP.

## 1. Introduction

Head and neck cancer encompasses a diverse group of malignancies arising from various anatomical subsites, e.g., the oral cavity, pharynx, larynx, or sinuses. Squamous cell carcinoma (SCC) is the most common histological subtype, accounting for 90% of cases [1]. Head and neck cancer of unknown primary (HNCUP) is defined as lymph node metastasis within the head and neck region in the absence of an identifiable primary tumor [2]. Following a comprehensive diagnostic evaluation, the primary tumor can be identified in up to 50% of cases of initial HNCUP, a considerable proportion (1.5–3%) remains unresolved, leading to uncertain treatment decisions [2,3]. About two-thirds of HNCUP cases are SCCs [4]. The most common site of HNCUP is the oropharynx (80–90%) [5].

Even though the primary tumor in HNCUP remains undefined by definition, in the majority of cases, cancer of unknown primaries (CUPs) can be explained by a distinct but small primary tumor. Nevertheless, CUPs can also be an entirely unique tumor entity [6,7,8]. The management of patients with HNCUP remains a diagnostic challenge, especially since there is no consensus about diagnostic procedures. Following a physical examination, a biopsy of the suspicious lymph node(s) and panendoscopy imaging is recommended. However, the choice of imaging modalities is still a matter of debate [2,9,10].

In this article, we aim to highlight the role of imaging using FAP-directed PET tracers, assess therapeutic implications, and compare this approach with conventional imaging techniques.

## 2. Conventional Imaging

Conventional imaging usually includes computed tomography (CT), magnetic resonance imaging (MRI), and ^18^F-Fluorodeoxyglucose positron emission tomography/computed tomography (FDG-PET/CT) [11]. Cross-sectional imaging with CT and MRI can detect up to 41% of HNCUPs [12]. Studies have demonstrated a higher detection rate for MRI compared to CT [12]. FDG-PET/CT is widely used in the diagnostic workup of HNCUP due to its ability to detect metabolically active lesions. PET/CT imaging is a hybrid imaging modality that combines functional (PET component) and anatomical information (CT component), thereby allowing precise localization and quantification of biological processes. The functional component is typically based on radiolabeled tracers targeting specific metabolic- or receptor-mediated pathways, while the CT component provides high-resolution anatomical detail [13]. FDG-PET/CT outperforms CT and MRI in identifying the primary site [12,14]. FDG-PET/CT shows a higher sensitivity (93% vs. 81%) and equal specificity (73%) compared to MRI, including diffusion-weighted imaging [15,16]. To date, FDG-PET/CT has been suggested to be the most sensitive imaging modality for the detection of HNCUP [17,18]. FDG-PET/CT not only helps to identify the primary, but also serves for staging purposes and, therefore, for treatment planning [19,20]. However, as FDG is not specific for malignancy, false-positive lesions may be detected, for example in the presence of inflammation or other benign conditions [21]. In addition, physiological FDG uptake can be seen in any lymphatic structure or neck muscle [12,22,23]. On the other hand, false-negative FDG uptake can be seen especially in small necrotic or well-differentiated metastases [24]. Therefore, novel tracers with improved detection rates of the primary in HNCUP are in demand.

## 3. FAPI-PET

### 3.1. Literature Review

Fibroblast activation protein (FAP) is overexpressed by cancer-associated fibroblasts in the microenvironment of various epithelial and mesenchymal malignancies, making them distinct from normal fibroblasts [25,26,27,28]. PET/CT using ^68^Ga- or ^18^F-labeled inhibitors of FAP (FAPI), such as [^68^Ga]Ga-FAPI-46 or [^18^F]F-FAPI-74, can visualize these cancer-associated fibroblasts. FAPI has been proven to be a particularly suitable tracer for the imaging of head and neck cancer (HN) with a high tumor-to-background contrast [29,30,31]. Kratochwil et al. presented a study with high uptake to HN lesions, as well as CUP lesions [32]. Several studies have shown that FAPI may be at least equivalent to FDG in terms of accuracy. In particular, the higher tumor-to-background ratios are superior when compared to FDG, despite a lower absolute signal intensity [33,34]. In a small cohort of twelve patients, FAPI and FDG both had a high sensitivity (100%) and accuracy (94.4%) in the detection of primary HN tumors or recurrence when validated with histopathological findings [35]. Gu et al. were the first to describe the diagnostic role of FAPI-PET/CT in HNCUP with negative FDG-PET/CT. In seven patients with a negative FDG scan, primary tumors were identified by FAPI-PET/CT. The primary sites included the nasopharynx, the palatine tonsil, the submandibular gland, and the hypopharynx. A total of 3/7 lesions were missed by FDG-PET/CT due to a lesion size < 10 mm [36]. A prospective comparative imaging study by Gu et al. compared the detection of HNCUP primaries between FDG and FAPI. Primary tumors were detected in 46/91 patients, with FAPI identifying more lesions than FDG (46 vs. 17; *p* < 0.001) with higher sensitivity and accuracy. The treatment was changed based on FAPI imaging results in 22/91 patients. The patients with unidentified primaries had a worse prognosis (hazard ratio, 5.77; 95% CI, 1.86–17.94; *p* = 0.0097), highlighting the diagnostic role of FAPI imaging in HNCUP [37].

Most of research focuses on SCC, since this is the predominant histological subtype [22]. Other histological subtypes, such as adenocarcinoma or neuroendocrine malignancies, are often not FDG-avid. Other tracers can, therefore, be used, but these usually target only one specific histological subtype, e.g., somatostatin-receptor-directed PET/CT [38]. In contrast, FAPI can assess a wide range of malignancies [32,39]. The American Society of Oncology guidelines recommend diagnostic tonsillectomy for patients with metastatic SCC of the neck and CUP [40]. Nevertheless, only 18–47% benefit from diagnostic tonsillectomy [41,42,43]. Serfling et al. showed that FAPI imaging led to a better detection of a primary located in the Waldeyer’s tonsillar ring, thus avoiding diagnostic tonsillectomy [44].

Accurate imaging of tumor lesions is particularly important for radiation planning, as inadequate radiation fields or radiation doses are a major cause of recurrence after radiotherapy [45]. Chen et al. presented a study showing that FAPI has a higher accuracy in assessing the N0 neck status compared to FDG (100% vs. 29%) [46]. There was only one study that reported an inferior detection rate of lymph node metastases by FAPI compared to that of FDG with histopathological confirmation [44]. Giesel et al. also demonstrated that FAPI-PET imaging provides a high tumor-to-background contrast. From a prospective clinical perspective, [^18^F]F-FAPI-74 PET appears particularly promising for precise radiotherapy planning. At the same time, it is associated with a lower radiation burden compared to that of [^18^F]FDG PET (0.020 vs. 0.024 mSv/MBq), further supporting its potential clinical advantage, especially in sensitive patient populations [47,48]. Table 1 provides an overview of the impact of FAP-directed imaging.

Diagnostic imaging can only influence patient management if it leads to a change in classification—either upgrading or downgrading the findings compared to conventional imaging—and thus changes in the therapeutic approach or decision-making process. To date, there is no evidence of upstaging of patients based on FAPI imaging alone, but FDG imaging must be interpreted with caution due to the potential false-positive uptake [39]. However, recent studies suggest that FAPI has superior diagnostic efficacy in the diagnosis of primary and metastatic disease [39]. Further radiotracers, especially hypoxia tracers, are currently only used for investigative purposes [49,50].

### 3.2. Case Study

**Figure 1:**
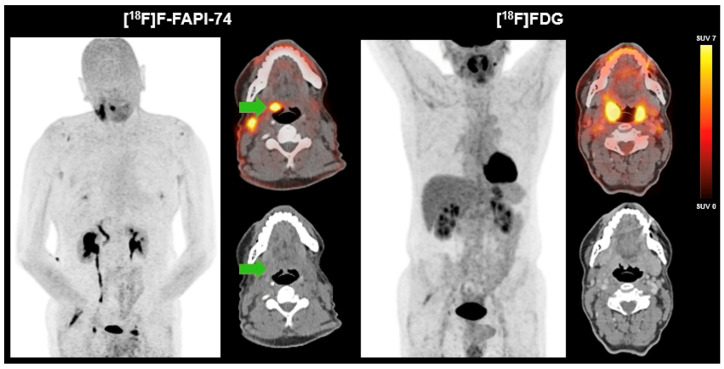
FAPI-PET/CT versus FDG-PET/CT in HNCUP.

We present a 60-year-old male patient who presented with a two-year history of right-sided cervical soft tissue swelling. MRI revealed a well-circumscribed, spherical lesion in the right mandibular angle with cystic-regressive components and strong contrast enhancement. Furthermore, multiple enlarged lymph nodes were observed bilaterally in the mandibular angle and along the vascular nerve sheath. The enhancement of contrast in the right lingual tonsil was deemed to be reactive. The preliminary differential diagnosis included lymphoma. Surgical excision of the lesion revealed a partially cystic-necrotic lymph node metastasis (maximum diameter 4.7 cm) of a moderately differentiated, keratinizing SCC without extracapsular spread. The presence of strong p16 expression was indicative of a probable association with human papilloma virus, with the oropharynx being identified as the presumed primary site. As no definitive primary tumor could be identified on initial imaging or clinical examination, the case was classified as HNCUP. FDG-PET/CT revealed symmetrical metabolic activity in the Waldeyer’s ring and a suspicious FDG-avid lymph node in the left upper vascular nerve sheath, but no distinct FDG-avid primary lesion. Subsequent [^18^F]F-FAPI-74-PET/CT demonstrated focal FAP expression in the right palatine tonsil, raising suspicion of a tonsillar carcinoma, as well as increased FAPI uptake in correlation with postoperative changes following lymph node dissection. In contrast to FAPI-PET/CT, no morphologically distinct lesion could be delineated in the region of the right palatine tonsil on CT. No further evidence of a FAP-expressing malignancy was detected (Figure 1). Definitive histopathology of the excised right tonsil and right tongue base confirmed infiltrates of a non-keratinizing, p16-positive SCC (pT2, pN2a [1/18, 4.7 cm], L0, V0, Pn0), with all surgical margins being negative (R0). 

In a 60-year-old male patient with HNCUP following lymph node excision of a partially cystic-necrotic lymph node metastasis of a moderately differentiated, keratinizing SCC without extracapsular spread, FDG-PET/CT showed no definitive evidence of a metabolically active primary tumor. There was diffusely increased but largely symmetrical FDG uptake in the Waldeyer’s ring and a single suspicious FDG-avid lymph node in the left upper cervical level along the vascular nerve sheath. In contrast, [^18^F]F-FAPI-74-PET/CT demonstrated focal, intense FAP expression in the right palatine tonsil (green arrow), suggestive of a primary tonsillar carcinoma. On CT, no morphologically distinct lesion could be delineated in the region of the right palatine tonsil, underlining the added diagnostic value of FAPI-PET in identifying the primary tumor site. Additionally, a diffuse FAPI uptake in the surgical bed following lymph node excision consistent with postoperative changes was detected. Definitive histopathology confirmed infiltrates of a non-keratinizing, p16-positive SCC.

## 4. FAP-Directed Theranostics

In comparison to FDG, FAPI-PET/CT can be further used to assess eligibility for FAP-directed radioligand therapy based on molecular-imaging-derived target expression. Due to a high tumor-to-background ratio, acceptable damage to the organs at risk may be assumed [51]. There are some data available on FAP-directed therapy with ^177^Lu or ^90^Y in different malignancies, however, the data for HN, or even HNCUP, are limited. Fu et al. presented a case report of a male patient diagnosed with non-keratinizing undifferentiated nasopharyngeal carcinoma with diffuse metastases of the lymph nodes and bones. Since the patient had no further treatment options, FAP-directed therapy with 3.7 GBq of [^177^Lu]Lu-FAPI-46 was conducted. The patient reported a decrease in ostealgia three days following the therapy, however, follow up imaging after eight weeks showed a mixed response by FAPI-PET/CT and progressive disease by CT. The patient declined further cycles of FAP-directed therapy [52]. Several studies showed an acceptable safety profile of FAP-directed therapy [53,54]. However, a prolonged circulation time of FAP-targeting antibodies and limited tumor retention were reported, since the radioligand binds to fibroblasts in the tumor microenvironment rather than to tumor cells themselves, in contrast to radioligand therapy with prostate-specific membrane antigens. The early wash-out has raised concerns about their therapeutic efficacy [55,56,57]. To overcome the prolonged circulation time of FAP-targeting antibodies, some recent studies have used FAP-targeting small molecules conjugated to radionuclides with shorter physical half-lives. The current research focuses on the further development of radioligands [58,59,60].

FAP-directed therapies have demonstrated objective responses in patients with advanced, treatment-refractory cancers. Nevertheless, larger-scale prospective studies are lacking, especially in HNCUP patients.

## 5. Conclusions

HNCUP remains a diagnostic and therapeutic challenge, with conventional imaging modalities often failing to identify the primary tumor. While FDG-PET/CT has improved sensitivity compared to MRI and CT, its limitations due to false positives and negatives necessitate alternative imaging approaches. FAPI-PET/CT has emerged as a promising modality, offering high tumor-to-background contrast, superior lesion detection (even in small or non-FDG-avid tumors), and potential impact on clinical decision making. Preliminary findings suggest that FAPI imaging may enhance primary tumor detection in HNCUP patients. Moreover, it holds potential for refining radiation planning by more accurately delineating tumor extent. In the future, FAP-directed radioligand therapy may offer a novel theranostic option for patients with widespread disease and high FAP expression. Despite encouraging results from retrospective cohorts and early prospective studies, large-scale prospective studies are needed to validate the diagnostic superiority and clinical impact of FAPI-PET/CT, particularly in the context of HNCUP. The integration of FAPI-PET/CT into diagnostic algorithms may be a decisive step towards precision imaging and personalized care for patients with HNCUP.

## Figures and Tables

**Table 1:** Overview of FAP-directed diagnostics.

Higher tumor-to-background ratio (FAPI vs. FDG)	[33,34]
Positive FAPI scan in patients with negative FDG scan is possible (higher sensitivity and accuracy)	[36]
Fewer diagnostic tonsillectomies due to improved primary detection with FAPI-PET/CT	[44]
FAPI-PET/CT is more accurate than FDG at assessing the N0 neck status (100% vs. 29%)	[46]

## Data Availability

No new data were created or analyzed in this study. Data sharing is not applicable to this article.

## Abbreviations

The following abbreviations are used in this manuscript:

CT	Computed tomography
CUP	Cancer of unknown primary
FAP	Fibroblast activation protein
FAPI	Fibroblast activation protein inhibitor
FDG	18F-Fluorodeoxyglucose
HN	Head and neck cancer
HNCUP	Head and neck cancer of unknown primary
MRI	Magnetic resonance imaging
PET	Positron emission tomography
SSC	Squamous cell carcinoma
